# Editorial: Interactive effects of climate change and human activities on plant productivity in grassland and cropland ecosystems

**DOI:** 10.3389/fpls.2026.1830378

**Published:** 2026-03-30

**Authors:** Yibo Li, Quanhui Ma, Junfeng Wang, Wenjiao Shi, Elvis F. Elli, Rogério de S. Nóia-Júnior

**Affiliations:** 1 Key Laboratory of Land Surface Pattern and Simulation, Institute of Geographic Sciences and Natural Resources Research, Chinese Academy of Sciences, Beijing, China; 2 College of Resources and Environment, University of Chinese Academy of Sciences, Beijing, China; 3 Ministry of Education Key Laboratory of Ecology and Resource Use of the Mongolian Plateau and Inner Mongolia Key Laboratory of Grassland Ecology and Observation and Research Station for the Typical Steppe Ecosystem of the Ministry of Education, School of Ecology and Environment, Inner Mongolia University, Hohhot, China; 4 Institute of Grassland Science, Key Laboratory of Vegetation Ecology of the Ministry of Education, Jilin Songnen Grassland Ecosystem National Observation and Research Station, Northeast Normal University, Changchun, China; 5 Department of Crop, Soil, and Environmental Sciences, University of Arkansas System Division of Agriculture, Fayetteville, AR, United States; 6 LEPSE, Univ Montpellier, INRAE, Institut Agro Montpellier, Montpellier, France

**Keywords:** climate change, cropland ecosystems, ecosystem multifunctionality, grassland ecosystems, human activities, plant productivity, sustainable management

## 1 Introduction

**Figure 1:**
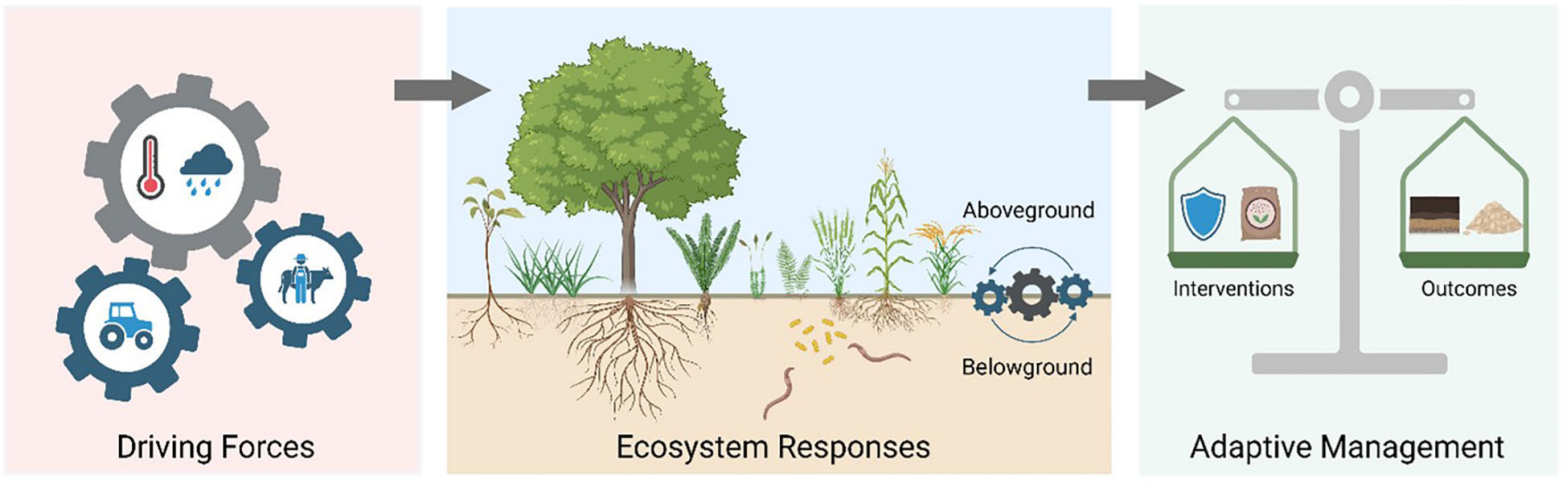
Conceptual framework of the Research Topic. The studies are organized into a causal chain from Driving Forces (climate change predictions and human disturbances) to Ecosystem Responses (mechanisms of biodiversity, soil processes, and physiology), and finally to Adaptive Management (strategies in grazing and cropland systems to enhance resilience and sustainability).

Grassland and cropland ecosystems constitute approximately 40% of the global terrestrial surface and provide essential services including food production, carbon sequestration, and biodiversity conservation (You et al., 2025). These ecosystems are increasingly subjected to the dual pressures of climate change—characterized by rising temperatures, altered precipitation patterns, and extreme weather events—and intensifying human activities such as agricultural management, grazing, and land-use transformation. While individual drivers have been extensively studied, their interactive effects introduce complex, often nonlinear dynamics that critically influence plant/crop productivity, community structure, and ecosystem functioning (**Figure 1**).

This Research Topic brings together 22 contributions spanning original research articles and systematic reviews that collectively advance our mechanistic understanding of climate-human activity interactions across diverse ecosystems. The contributions employ complementary methodological approaches, from long-term field experiments and manipulative studies to remote sensing analyses, meta-analyses, and machine learning-based predictions, and cover geographical regions from the Qinghai-Tibet Plateau to the North China Plain, from Central Asian drylands to Turkish highlands. Together, these contributions provide new insights into how climate change and human activities jointly influence plant productivity and ecosystem functioning in grassland and cropland systems. The following sections synthesize the main findings across key thematic areas represented in this Research Topic.

## 2 Grassland management and community dynamics

A substantial portion of this Research Topic addresses how management practices shape grassland ecosystem structure and function. Shi et al. conducted a three-year observational study in the Hulunbuir temperate meadow steppe, China, demonstrating that enclosure enhances plant diversity and community stability more effectively than grazing and mowing through strengthened species turnover regulation and soil processes. Their structural equation modeling revealed that soil bulk density, total nitrogen, and organic carbon were key environmental factors mediating diversity-stability relationships. Complementing this work, Mi et al. compared grazed and non-grazed steppe ecosystems across three typical grassland sites in Inner Mongolia, finding that grazing reshaped ecosystem regulation by inverting soil nutrient-biomass relationships and shifting microbial community control from dual nutrient-pH regulation to pH-dominated patterns.

The meta-analysis by Zheng et al. synthesized 73 peer-reviewed studies evaluating grazing intensity impacts across five main grassland types in Xinjiang, revealing that heavy grazing significantly reduced soil organic carbon while moderate grazing showed threshold effects depending on ecosystem context. Xin et al. examined the interactive effects of rainfall increase and nitrogen deposition on a *Leymus chinensis* steppe during postgrazing succession, finding no synergistic effects, a result challenging the assumption about resource co-limitation. The systematic review by Yang et al. synthesized global data on grassland root decomposition, concluding that litter quality, particularly the ratio of acid-unhydrolyzable residue to nitrogen, outweighs climate as the dominant driver of root mass decomposition.

## 3 Cropland management, soil carbon, and sustainable intensification

Several contributions addressed the critical nexus of agricultural management, soil health, and carbon cycling in cropland systems. Wang et al. presented insights from a 20-year on-farm observational experiment of a winter wheat-summer maize rotation in the North China Plain, documenting sustained yield improvements alongside divergent carbon dynamics. The authors found the wheat season transitioned into a net carbon sink due to SOC accumulation from straw incorporation, while SOC depletion during the maize season from surface mulching resulted in a carbon source status. Wang et al. demonstrated that subsoiling enhanced soil organic carbon and ecosystem multifunctionality in fluvo-aquic soils, offering practical guidance for sustainable intensification. Xie et al. investigated an approach combining a chemical growth promoter with canopy top removal to simultaneously enhance maize lodging resistance and yield, achieving 8-9% yield increase while reducing lodging by 70-81%.

## 4 Climate change projections and species distribution modeling

Multiple contributions employed climate projections and species distribution models to assess future risks. Xu et al. applied bias-corrected CMIP6 projections to investigate hydroclimatic evolution in Xinjiang under three SSP scenarios, revealing significant warming trends, increased potential evapotranspiration and widespread aridification. Chen et al. examined projected changes in extreme climate indices affecting rice production, demonstrating that extreme heat events in southern China will increase by 12–18 days per year by the 2080s under SSP5-8.5.

Species distribution modeling studies have provided critical insights into climate change impacts on plant ranges. Abay and Gül, Abay and Gül modeled the endemic Turkish moss *Cinclidotus bistratosus*, identifying precipitation as a key distribution driver and projecting substantial habitat decline under warming scenarios. Their companion study on 17 *Sphagnum* species similarly projected distribution contractions. Using optimized Maximum Entropy (MaxEnt) modeling to assess ecological niches, Chen et al. predicted habitat suitability for the medicinal plant *Verbena officinalis*, projecting habitat expansion with significant northward shifts under warming climates.

## 5 Ecosystem services, multifunctionality, and trade-offs

Understanding ecosystem services and their trade-offs emerged as a central theme. Du et al. applied an XGBoost-SHAP machine learning methodology to quantify nonlinear effects and threshold responses of ecosystem service trade-offs in ecologically fragile areas, with land use type, precipitation, and temperature as dominant drivers. Pang et al. conducted a spatiotemporal interaction analysis of ecosystem health and human activity intensity in the Daxing’anling forest-grassland ecotone, revealing a significant negative spatial correlation (Moran’s I = -0.62), highlighting development-conservation trade-offs. Wang et al. revealed contrasting relationships between biodiversity and ecosystem multifunctionality in subtropical forests: plant phylogenetic diversity promoted multifunctionality while soil bacterial diversity showed an opposite trend.

## 6 Vegetation dynamics and remote sensing applications

Advances in remote sensing provided new tools for monitoring vegetation responses across scales.

Sun et al. analyzed vegetation dynamics in the Qinghai Lake Basin, China, using MODIS-EVI data, reporting a significant annual increase (1.38 ×10^-3^) in fractional vegetation cover in the Qinghai Lake Basin, China. They demonstrated that temperature and precipitation synergistically promote growth, but actively suppress it when exceeding their optimal ranges (-6 to 0 °C and 325–550 mm). Li et al. examined global soil moisture dynamics and their association with gross primary productivity, finding strong positive correlations in the 10–100 cm root zone of grasslands and croplands. To estimate aboveground biomass in savanna grassland, Chen et al. integrated remote sensing, meteorological, topographic, and biodiversity variables into a Random Forest model, achieving an *R*^2^ of 0.70. Xiao et al. explored utility-scale solar energy expansion, revealing ecological dichotomies between arid and humid regions. In arid Ningxia, photovoltaic installations had negligible effects on vegetation greenness, while in humid Anhui, NDVI declined by over 50%. These findings advocate for strategic prioritization of arid regions with greater ecological resilience for renewable energy development.

## 7 Plant stress responses and plant-microbe interactions

Two contributions examined plant-microbe interactions under environmental stress. Song et al. investigated bacterial community dynamics in *Allium senescens* under drought stress, finding that drought reduced rhizosphere bacterial diversity by 42% while increasing leaf endophyte diversity by 52%, with *Streptomyces* and *Ralstonia* enriched under drought conditions. Ji et al. integrated species distribution modeling with phytochemical analysis for the medicinal holoparasitic plant *Cynomorium songaricum*, identifying precipitation and soil pH as key habitat drivers while revealing that slope, minimum temperature, and isothermality regulate bioactive component accumulation.

## 8 Conclusions and future perspectives

The contributions to this Research Topic advance our understanding of climate-human activity interactions in several important ways. First, they demonstrate pronounced context-dependency: effects that are beneficial in one ecosystem type may be detrimental in another, as illustrated by the contrasting grazing responses across grassland types and the ecological dichotomies of solar energy expansion. Second, they reveal threshold effects and nonlinear responses that require moving beyond simple linear driver-response frameworks, particularly evident in the XGBoost-SHAP analyses. Third, they highlight the importance of belowground processes—root decomposition, soil carbon dynamics, and plant-microbe interactions—that critically mediate ecosystem responses to environmental changes.

Looking forward, several research priorities emerge. There is a pressing need for long-term, multi-factorial experiments that can capture interactive effects and temporal lags in ecosystem responses. The integration of mechanistic understanding with machine learning approaches offers promising avenues for improving predictions across scales. Bridging the grassland-cropland divide to identify general principles of ecosystem resilience remains an important challenge. Finally, translating scientific understanding into actionable management recommendations requires continued multidisciplinary collaboration among researchers, land managers, and policymakers.
